# Translation, cultural adaptation and validation of the Swahili Pain Catastrophizing Scale among refugees who survived torture and/or war trauma in Kenya: An observational study

**DOI:** 10.1002/hsr2.2095

**Published:** 2024-05-16

**Authors:** Jepkemoi J. Kibet, Joliana S. Phillips, Mariem C. Latrous, Hanan Khalil, Linzette D. Morris

**Affiliations:** ^1^ Department of Physiotherapy University of the Western Cape Bellville South Africa; ^2^ Department of Rehabilitation Sciences, College of Health Sciences, QU Health Qatar University Doha Qatar

**Keywords:** cross‐cultural adaptation/validation, Kenya, pain catastrophization scale, refugees, survivors of torture

## Abstract

**Background and Aims:**

Accurate assessment of any patient relies on the use of appropriate measurements which are culturally‐ and linguistically‐applicable and valid. The following study aimed to translate, cross‐culturally adapt and test the nomological validity, structural validity, internal consistency, test‐retest reliability, sensitivity‐to‐change and feasibility of the Swahili version of the Pain Catastrophizing Scale (Swa‐PCS) among refugees who survived torture/war trauma living with chronic pain in Kenya.

**Methods:**

An observational study was conducted. Translation and cultural adaptation of the original PCS for the Swahili‐speaking refugee population in Kenya, who survived torture or war trauma was undertaken. Following this process, a validation study was conducted on the newly‐adapted instrument, to ascertain the psychometric properties (nomological validity, structural validity, internal consistency, test‐retest reliability, sensitivity‐to change, and ceiling and floor effects).

**Results:**

Fifty participants were included in this study. Correlations between pain catastrophization and fear‐avoidance behavior measures were significant (*r* = 0.538, *p* < 0.01). Ceiling effects were 42−48% with no floor effects. Standard errors of measurement values were between 0.938 and 3.38. Minimal‐detectable‐change values were between 2.17 and 7.82. Internal consistency was satisfactory to good, for the whole and subsections respectively (range *α* = 0.693−0.845). Magnification had the lowest *α*. Test−retest reliability was also satisfactory to good (range ICC =  0.672−0.878). Confirmatory factor analysis confirmed that the Swa‐PCS had three factors which explained the majority of the variance. Root mean square error of approximation and comparative fit index were calculated for goodness‐of‐fit assessment, and were 0.18 and 0.83, respectively.

**Conclusion:**

This study showed that the adapted Swa‐PCS displayed overall satisfactory to good internal consistency, test‐retest reliability and sensitivity‐to‐change. Furthermore, the Swa‐PCS scores were related to fear‐avoidance behavior scores as expected (nomological validity). Structural validation of the Swa‐PCS requires further investigation. Further testing of the psychometric properties of the Swa‐PCS is however warranted.

## 1 INTRODUCTION

Every day, numerous people around the world are forcibly displaced from their home countries and become refugees due to ongoing wars, conflict and political unrest.^1^, ^2^ Over the past few decades, the world has witnessed an unprecedented increase in the number of refugees seeking asylum in foreign countries for a better and safer life. Nevertheless, refugees are typically exposed to various traumatic and stressful experiences that is, loss of property/land, loss and separation from family, lack of access to food/water, inadequate access to health care, physical/sexual assault, violence, and torture.^3^, ^4^ Abu Suhaiban et al.^3^ reports that approximately 35% of refugees have experienced some sort of torture.

Refugees, and especially those who are survivors of torture, are particularly at high risk of developing chronic pain.^5^, ^6^ It has been proposed that following traumatic experiences, a “generalized dysfunctional pain modulation” may be provoked within the individual's pain physiology, which may trigger intense chronic pain.^7^, ^8^ Essentially, in these individuals, the body's ability to modulate and regulate pain responses becomes disrupted, leading to heightened sensitivity to pain stimuli and altered pain processing mechanisms, which manifests as an exaggerated and widespread perception of pain, extending beyond the site of injury or trauma.^8^ This may explain why up to 92% of people who have experienced torture or trauma present with chronic pain, especially painful conditions of the musculoskeletal system.^6^, ^9^

How well a person copes with their chronic pain is however largely dependent on the coexistence of various psychological factors that is, pain catastrophizing,^10^ kinesiophobia^11^, ^12^ pain acceptance^13^ and pain vigilance.^14^, ^15^ Of these psychological factors, pain catastrophizing is the main factor identified as predicting adjustment to chronic pain and is a significant moderator of chronic pain.^10^, ^16^, ^17^ Defined as a “negative and exaggerated response to a given painful stimulus,”^18^ pain catastrophization is associated with functional disability, pain severity and depression in patients with chronic pain.^18^, ^19^ Pain catastrophization amplifies perception of pain and emotional distress, and therefore leads to increased disease activity, prolonged episodes of pain and fear avoidance behaviors.^18^, ^19^ Evidence suggests that pain catastrophizing has a consistently strong correlation with pain severity, disability, performance of activities of daily living and mood among people with chronic pain.^13^, ^20^, ^21^ In an already vulnerable population, like refugees, this interconnecting effect of pain, catastrophization and disability can be detrimental to the individual's functioning, well‐being and adjustment to their new environment. Therefore, of particular interest in this population, is the role that pain catastrophization plays in the link between trauma, pain and disability.^5^ However, before effectively managing pain catastrophization in refugees who have survived of torture and/or war trauma to prevent further disability, it is imperative that the level of pain catastrophization is accurately measured using appropriate and valid outcome measures. To date, the most common outcome measurement tool used to measure pain catastrophization is the pain catastrophizing scale (PCS), developed by Sullivan et al. in 1995.^18^

The PCS, a self‐administered questionnaire which consists of 13 items and 3 subscales: Helplessness, Magnification and Rumination, has been demonstrated to be a helpful indicator of pain catastrophizing across a range of pain conditions.^18^ The validity and reliability of the English version of the PCS has been investigated extensively, and its psychometric properties have been reported as very good.^18^, ^22^ The PCS is therefore a reliable and valid tool to assess the impact of catastrophizing on the experience of pain^18^, ^22^ and is a widely used measure for pain catastrophization in clinical practice and research. Over the years, the PCS has been translated into many languages and cross‐culturally adapted for various populations i.e. Hausa, Hindi, Catalan, Korean, German, French‐Canadian, Argentine, Bengali, South African, Greek and Malay.^23^, ^24^, ^25^, ^26^, ^27^, ^28^, ^29^, ^30^, ^31^, ^32^, ^33^

However, to our knowledge, the PCS has not been validated among any refugee population in the world. In addition, there is currently no Swahili version of PCS which can be used among a refugee population in Kenya, a country known to host many refugees from the East African region where Swahili is a widely‐spoken language.^34^ The purpose of the following study was therefore to translate, cross‐culturally adapt and test the nomological validity, structural validity, internal consistency, test‐retest reliability, sensitivity‐to‐change and ceiling/floor effects of the adapted Swahili‐version of the PCS among Swahili‐speaking refugees who were survivors of torture and/or war trauma attending the “REDACTED” situated in Nairobi city, Kenya. A validated and reliable Swahili version of the PCS could potentially improve the accuracy of measurement among this population and thereby enhance the quality of health care provided to the refugee population in Kenya. Research among refugees in the future can also be facilitated.

## 2 METHODS

The following cross‐sectional, observational study was reported based on the Strengthening the Reporting of Observational studies in Epidemiology (STROBE) guidelines.^35^, ^36^

### 2.1 Ethical clearance

The University of the Western Cape (South Africa) Senate Research Ethics Committee(s) approved this study (Registration no.15/3/21). Local ethical approval was also obtained from the Institutional Research and Ethics Committee (IREC) situated at the Centre for Victims of Torture (CVT) in Kenya. Permission to conduct the study was granted by the Director of Research at the CVT, an international Non‐Governmental Organization (NGO) in Nairobi, Kenya. The CVT did not actively participate in the research, but provided a site for conducting the study. All eligible subjects participated voluntarily and confidentially, and were provided with an extensive explanation of the study procedure in Swahili. Informed consent was read to the participants individually, and was signed in the Swahili language. The study was conducted adhering to the ethical principles described in the Declaration of Helsinki.

### 2.2 Study population and recruitment process

Eligible subjects were conveniently sampled from the clients referred to physiotherapy at the CVT. The CVT is dedicated to providing holistic health care to survivors of torture which includes trauma‐focused physiotherapy, mental health counseling and social services. Contact information was retrieved from the original referral forms which were filed and kept in a locked metallic cabinet in an access‐controlled room at the CVT offices. All attempts to reduce bias and maintain confidentiality was made since the principal researcher worked at the CVT. Clients were not anonymous to the principal researcher, but their data remained confidential and was not made public to unauthorized persons. Eligible subjects were contacted telephonically and were included if they were: male or female adults aged 18 years and older, refugees referred to physiotherapy services at CVT in Nairobi, Kenya, survivors of torture and/or war trauma, suffered from chronic nonmalignant musculoskeletal pain (pain persisting ≥ 6 weeks) at the spine and/or any part of the body, their telephonic contact details were available, they were sufficiently proficient in Swahili and they consented to participate in this study. Language proficiency and comprehension of what was expected from the participant was assessed by the principal researcher.

### 2.3 The PCS instrument and other data collection tools

A specifically‐designed sociodemographic form was used to collect sociodemographic data and other information. The original PCS is a self‐administered questionnaire with 13 items and 3 subscales: Helplessness (items 1−5 and 12), Magnification (items 6, 7, and 13) and Rumination (items 8−11), and has shown to have a high internal consistency, Cronbach alpha (*α*) = 0.87.^18^ Using a 5‐point Likert scale for each question, patients are requested to rate to which degree they experience the thoughts stated, from 0 (never) to 4 (always).^37^ The highest possible score for the PCS is 52, with higher scores indicating higher levels of pain catastrophization. The following outcome measures were also included: (a) the visual analogue scale (VAS)^38^ and (b) the Fear Avoidance Belief Questionnaire (FABQ). The VAS consists of a continuum line measuring 100 mm and is a widely used measure of pain. Consistently reported to be a valid and reliable tool, the VAS is used in clinical and research globally.^39^ According to the results of a critical review conducted by,^39^ the VAS has high test‐retests reliability and repeatability. The maximum score on the VAS is 100, and the higher the score the higher the pain measurement. The FABQ is a 16 item, two‐factor self‐reported questionnaire which collects data on the respondents' belief about “how physical activity and work affect their pain.”^40^ The internal consistency of the FABQ has been reported as good, with Cronbach's α ranging from 0.75 to 0.82.^41^, ^42^ The highest possible score for the FABQ is 96, and the higher the score the higher the fear‐avoidance behavior index. For the purposes of this study, the FABQ and VAS were translated to the Swahili language alongside the PCS, but no psychometric testing was conducted.

### 2.4 Study procedure

This cross‐sectional study consisted of two phases. Firstly, the translation and cultural adaptation of the original PCS for a Swahili‐speaking refugee population in Kenya was undertaken. Secondly, a validation study was conducted to ascertain the psychometric properties (viz. nomological validity, structural validity, internal consistency, test‐retest reliability, sensitivity‐to‐change and ceiling/floor effects) of the newly‐adapted Swahili instrument among this population.

### 2.5 Linguistic and cultural adaptation process of the PCS

The original developer of the scale was contacted via email for permission to translate and adapt the PCS into a Swahili version (Swa‐PCS). The translation procedure was aimed at achieving semantic and conceptual equivalence between the original English PCS and the Swahili version of the PCS and followed guidelines outlined by Beaton et al.^43^ and World Health Organization (WHO).^44^ The original PCS was sent to two professional, freelance Swahili translators with no prior knowledge of the original version. The translators were also not aware of the concepts being investigated, did not have a medical background, and aimed to identify inconsistencies, cultural diversities, conceptual equivalences, and variances in vocabulary. Translations were performed independently and discrepancies were discussed and resolved. The translators synthesized their forward translations and provided a written report listing how issues were resolved. Independent and blinded back translations of the Swahili PCS into English were performed by two independent translators. Back translations were compared with the original PCS. The translators ensured that standard Swahili was used to ensure equivalence between the original English PCS and the Swahili version.

For the cultural adaptation process, a cross‐cultural expert committee consisting of two physiotherapists and two occupational therapists, who were all bilingual and worked in Kenya's health care system, were invited to participate. A subgroup of 10 Swahili‐speaking refugees attending the CVT were also invited to participate in this process. The Swahili version of the PCS was sent to the expert committee members via e‐mail and was administered to the subgroup of refugees during a scheduled appointment. Both groups were asked to review the translated PCS, comment if there were any items in the translated version which are not applicable to the context, and provide relevant suggestions to render the questions more applicable to the proposed population. The principal researcher collated the suggested changes per aspect of the questionnaire (i.e., instruction, wording of items, structure, and scoring system). The expert committee checked the adapted PCS for semantics, idiomatic and conceptual equivalence. All modifications were approved by the expert committee and the prefinal version of the Swahili‐PCS (Swa‐PCS) was produced.

### 2.6 Pretesting of the Swa‐PCS version: Face equivalence and content validity

The pre‐final version of the Swa‐PCS was field tested among a convenient sample of 20 individuals (Swahili‐speaking refugees), older than 18 years old, attending physiotherapy. Face equivalence/validity and content validity were qualitatively assessed. Face validity is defined as “whether the items of each domain are sensible, appropriate, and relevant to the people who use the measure on a day‐to‐day basis.”^45^ Content validity is defined as “the extent to which the set of items comprehensively covers the different components of health to be measured.”^45^ The time taken to complete the adapted PCS was recorded.

### 2.7 Data management and statistical analysis

Incomplete forms, or forms completed incorrectly, were considered but discarded if necessary. No weighting of items was used, and no imputation of missing data was deemed necessary. Data collected were extracted and entered into a purpose‐built MS Excel worksheet, and exported to SPSS version 25.0 statistical software.^46^ Data related to the socio‐demographic information, chronic pain symptoms, etc. were analyzed accordingly using descriptive statistics. Level of significance was set a priori at 0.05, with tests for significance being two‐sided. Psychometric testing for validity and reliability of the adapted measure included nomological validity, structural validity, internal consistency, test−retest reliability, sensitivity‐to‐change and ceiling and floor effects.

### 2.8 Validity testing: Validity of the Swa‐PCS was assessed through nomological and structural validity

#### 2.8.1 Nomological validity

Nomological validity, also known as external validity, is a form of construct validity which tests the relationship between constructs which are expected to be linked.^47^ Pearson's correlation coefficients (*r*) were calculated for correlations between pain catastrophization (PCS scores), fear‐avoidance behaviors (FABQ scores), and pain intensity (VAS scores) to ascertain nomological validity between these constructs of interest. The hypotheses were that the PCS scores would correlate significantly (*p* < 0.05) with the FABQ, but most likely not with the VAS scores.

#### 2.8.2 Structural validity

Structural validity of the adapted PCS was assessed using confirmatory factor analysis with maximum likelihood estimation and was conducted using only the questionnaires without missing data. Confirmatory factor analysis was undertaken since it was pre‐specified that the observed variables could be explained by the underlying factors or constructs.^48^ For the confirmatory factor analysis, a varimax oblique rotation model was used and scree plots of eigenvalues were generated. Item loadings of ≥0.4 were considered to be included in a component.^49^ Root mean square error of approximation (RMSEA) and comparative fit index (CFI) were calculated for goodness‐of‐fit assessment. RMSEA values < 0.10 and CFI values > 0.95 were considered a good fit.^49^ The purpose of this analysis was to ascertain how many factors could explain the variance in the data and to evaluate model fit. The three‐factor model used was determined a priori based on the following dimensions identified in the original PCS—*Helplessness, Magnification* and *Rumination*.^18^ However, before conducting the confirmatory factor analysis, suitability of performing factor analysis was ascertained through the Bartlett's test of Sphericity and the Kaiser−Meyer−Olkin (KMO) tests which measure sampling adequacy.^48^ To be considered for factor analysis, the Bartlett's test of Sphericity had to be significant (*p* <0.05). The KMO index ranges from 0 to 1 and the index can be interpreted as follows: 0.8 or above, as meritorious; 0.7 or above, as middling; 0.6 or above, as mediocre; 0.5 or above as miserable; and below 0.5 as unacceptable.^48^

### 2.9 Reliability testing: Reliability of the Swahili PCS was established by means of internal consistency, test‐retest reliability, ceiling and floor effects, and sensitivity‐to‐change

#### 2.9.1 Internal consistency

According to DeVon et al.^50^ internal consistency “relates to the homogeneity of the scale and how well items on a tool fit together.” The internal consistency of the final cross‐culturally adapted and translated Swa‐PCS (whole and subsections) was estimated using Cronbach's alpha (*α*) from 0 to 1. The Cronbach *α* values between 0.60 and 0.8 represents acceptable to satisfactory internal consistency, above 0.8 represents very good internal consistency, and above 0.9 excellent internal consistency.^51^

#### 2.9.2 Test−retest reliability

Participants included in the reliability testing of this study were asked to complete the Swa‐PCS at 2‐points in time, 2 weeks apart. Test‐retest reliability measures stability and reproducibility of a measure over time, and is relevant for scales which measure cognitive and trait information which are not expected to change in short period of time.^52^ To evaluate test‐retest reliability, intraclass correlation coefficients (ICC) and 95% confidence intervals (CIs) were calculated for the Swa‐PCS (whole and subsections). The ICC is “an index of the reliability of the measurements between tests.”^53^ ICC values of 0.6 to 0.8 were regarded as evidence of acceptable to good reliability, higher than 0.8 were considered as very good to excellent reliability.^53^

#### 2.9.3 Ceiling and floor effects

Sensitivity and ability to distinguish between respondents was established by calculating the floor and ceiling effects and considered if ≥15% of the participants scored the highest or lowest scores.

#### 2.9.4 Sensitivity‐to‐change

To ascertain the Swa‐PCS’ sensitivity‐to‐change, various measurement errors were calculated.^54^ Sensitivity‐to‐change is defined as “the capacity of a measure to detect change in patients over time” and relates to the “clinical meaningfulness of changes in scores.”^54^ The following measurement errors were calculated: standard error of measurement (SEm), limits of agreement/Bland Altman plot and minimal detectable change (MDC).^55^ A SEm “estimates the variation around a “true” score for an individual when repeated measures are taken.”^55^ In addition, agreement between the baseline and retest assessment was illustrated using Bland and Altman plots which were used to calculate the 95% limits of agreement.^56^

## 3 RESULTS

### 3.1 Linguistic and cultural adaptation

Following the expert committee review, several modifications were made to the overall layout/structure, the instructions on how to complete the PCS, the scoring system and wording of the items to make the PCS more culturally applicable for refugee survivors of torture with chronic pain living in Nairobi, Kenya (Table 1).

**Table 1:** Changes made to various components of the original PCS.

Component of original PCS	Modifications made to produce the Swa‐PCS
Layout and structure	− Tick boxes were placed next to each question. − The section related to personal information was removed and replaced with a section for the client study code number and date.
Instructions	Instructions were revised and simplified.
Anchors and Scoring system	The anchors “not at all,” “to a slight degree,” “to a moderate degree,” “to a great degree,” and “all the time” were retained in the adapted version of the PCS.
Scoring system	The score for each anchor remained the same as the original PCS, ranging from “0” for “not at all” to “4” for “all the time”.
Wording of items	− A number of changes were made to the wording of the items based on the suggestions made by the expert committee and the client subgroup improve the cultural appropriateness. − To facilitate understanding, the phrase “when I am in pain” which related to the experience of being in pain/moment in pain, was added to each item. − The phrase “when I am in pain, I feel I can't go on “ in item two was difficult to translate into Swahili. The translators therefore agreed to use the word “continue” for easy understanding.

#### 3.1.1 Sample characteristics

Of the 72 contact details received, 69 refugees were contactable by phone and invited to participate in the study. Following eligibility screening, 25 participants were excluded and another three participants declined the invitation. Fifty‐five participants were initially included, however five did not complete the forms correctly, and their data were excluded in the analyses. Fifty participants were included in the final analyses. Table 2 depicts the sociodemographic information of the included subjects. The types of torture endured by the included participants of this study varied from sexual assault and/or physical abuse. Table 3 depicts the data recorded relating to the included subjects' pain experiences (area of pain, pain intensity).

**Table 2:** Subject sociodemographic information (*n* = 50).

**Variable**	***N* (%)**	**Mean (SD)**
Gender		
Female	29/50(58%)	
Male	21/50 (42%)	
Age—in years (year)		
All	50/50 (100%)	31 year (8.80)
Female	29/50 (58%)	31 year (10.08)
Male	21/50 (42%)	30 year (6.84)
Education		
None	0/50 (0%)	
Primary	6/50 (12%)	
High school	17/50 (34%)	
Tertiary	27/50 (54%)	
Marital Status		
Single	17/50 (34%)	
Married	27/50 (54%)	
Divorced	0/50 (0%)	
Widowed	4/50 (8%)	
Separated	2/50 (4%)	
Home country		
Burundi	4/50 (8%)	
Eritrea	2/50 (4%)	
Ethiopia	1/50 (2%)	
Democratic Republic of Congo	29/50 (58%)	
Somalia	8/50 (16%)	
Uganda	6/50 (12%)	

**Table 3:** Pain and other outcomes for included subjects (*n* = 50).

Variable	*N* (%)	Mean (SD)
Years since torture 8 ‐ in years (y)		
All	50/50 (100%)	3 y (1.69)
Female	29/50 (58%)	3 y (1.71)
Male	21/50 (42%)	2 y (0.67)
Area of most severe pain		
Head	8/50 (16%)	
Neck	3/50 (6%)	
Upper limbs	12/50 (24%)	
Back	19/50 (38%)	
Abdomen	1/50 (2%)	
Lower limbs	6/50 (12%)	
Pain intensity (VAS 100 mm scores)		
All	50/50 (100%)	82.08 (11.59)
Female	29/50 (58%)	83.59 (9.86)
Male	21/50 (42%)	80.03 (13.60)
Fear‐avoidance behavior (FABQ scores)		
All	50/50 (100%)	77.62 (8.34)
Female	29/50 (58%)	77.62 (8.34)
Male	21/50 (42%)	77.37 (8.44)
Pain catastrophization (PCS scores)		
All	50/50 (100%)	49.12 (4.11)
Female	29/50 (58%)	48.25 (7.43)
Male	21/50 (42%)	48.13 (7.29)

#### 3.1.2 Pretesting results: Face equivalence and content validity

All the participants who participated in the face equivalence and content validity phase felt that the questions were clear and easy to understand. No further changes/modifications were deemed necessary and the subjects were in agreement with the layout/format of the adapted questionnaire. Most participants “agreed” that the Swa‐PCS was easy to complete and all participants deemed all the items important and applicable. The mean (SD) time taken to complete the questionnaires was 6.04 (2.2) min. The final version of the Swa‐PCS is provided in Appendix 1.

### 3.2 Validity

#### 3.2.1 Nomological validity

Correlations between the PCS and FABQ scores were significant (*p* < 0.05) for both time points, time A (*r* = 0.33, *p* < 0.01*) and time B* (*r* = 0.54, *p* <0.001). No significant correlations were found between the PCS and VAS scores at time 1 (*r* = 0.23; *p* = 0.11) and neither for FABQ scores and VAS scores (*r* = 0.06; *p* = 0.67). The VAS scores were only collected at the first visit and were not correlated for both time points. The hypothesis that the PCS scores would be significantly correlated with the FABQ scores was met.

#### 3.2.2 Structural validity

The KMO value was 0.714 and the Bartlett's Test of Sphericity was significant (*X* ^2^ = 610.92; *p* < 0.001), indicating that the sample was adequate and that confirmatory factor analysis was appropriate.^48^ Examination of the scree plot of eigenvalues confirmed that the “elbow” of the graph was visible around the 3rd factor, indicating that there were three major factors which could explain the variance in this data set and should be retained. The three components explained 74.51% of the variation as follows: component 1 (helplessness) = 49.17%; component 2 (magnification) = 16.65% and component 3 (rumination) = 8.69%. All items had high factor loading on one or more components, and ranged from 0.49 to 0.96 (Table 4). Based on the three varimax factor model (3 + 4 + 6 items) and using a maximum likelihood estimation, the *X* ^2^ = 121.79 (*df* = 42), the RMSEA was calculated as 0.18 and the CFI as 0.83. Item analysis for each item in the PCS is provided in Table 5.

**Table 4:** Factor loading based on Confirmatory factor analysis of the Swahili‐PCS.

	Component			
	1 Helplessness	2 Magnification	3 Rumination	Communalities
Item 3	**0.956**	0.122	−0.021	0.930
Item 2	**0.897**	0.108	0.237	0.873
Item 4	**0.894**	0.213	−0.109	0.857
Item 5	**0.869**	0.189	0.094	0.800
Item 1	**0.743**	0.005	0.279	0.630
Item 13	**0.670**	0.116	**0.537**	0.750
Item 7	**0.652**	**0.527**	0.241	0.760
Item 8	0.135	**0.841**	−0.138	0.745
Item 9	0.101	**0.717**	**0.488**	0.863
Item 11	0.039	**0.711**	0.159	0.532
Item 6	**0.554**	**0.690**	−0.096	0.792
Item 10	0.135	**0.669**	**0.498**	0.713
Item 12	0.096	0.092	**0.723**	0.541

*Note*: The bold indicates where factors had the highest loading.

**Table 5:** Item analysis of PCS (first and second administration).

First administration of PCS						Second administration of PCS					
PCS Item	Mean	SD	95% CI	Skewness	Kurtosis	PCS Item	Mean	SD	95% CI	Skewness	Kurtosis
**1**	3.64	0.75	3.43‐3.85	−2.93	10.87	**1**	3.70	0.61	3.53‐3.87	−2.48	7.20
**2**	3.76	0.48	3.62‐3.89	−1.90	2.66	**2**	3.72	0.67	3.53‐3.91	−3.82	18.9
**3**	3.76	0.56	3.06‐3.92	−3.03	11.63	**3**	3.62	0.70	3.42‐3.82	−3.09	13.91
**4**	3.82	0.39	3.71‐3.93	−1.72	0.99	**4**	3.62	0.70	3.42‐3.82	−3.09	13.91
**5**	3.70	0.51	3.56‐3.843	−1.39	0.98	**5**	3.62	0.72	3.41‐3.83	−2.61	11.67
**6**	3.80	0.40	3.69‐3.91	−1.55	0.41	**6**	3.72	0.45	3.59‐3.85	−1.01	−1.02
**7**	3.80	0.45	3.67‐3.93	−2.21	4.48	**7**	3.72	0.57	3.56‐3.88	−1.29	1.81
**8**	3.84	0.37	3.73‐3.95	−1.91	1.73	**8**	3.76	0.43	3.64‐3.88	−1.26	−0.44
**9**	3.76	0.52	3.61‐3.91	−2.13	3.89	**9**	3.78	0.46	3.65‐3.91	−2.01	3.47
**10**	3.78	0.42	3.66‐3.89	−1.40	−0.61	**10**	3.84	0.37	3.73‐3.95	−1.91	1.73
**11**	3.78	0.47	3.65‐3.91	−2.01	3.47	**11**	3.80	0.45	3.67‐3.93	−2.21	4.47
**12**	3.78	0.51	3.64‐3.92	−2.32	4.77	**12**	3.72	0.54	3.57‐3.87	−1.81	2.51
**13**	3.90	0.30	3.81‐3.99	−2.75	5.80	**13**	3.78	0.65	3.59‐3.96	−3.51	12.68
**Total**	**49.12**	**4.1**	**47.95‐50.29**	**−1.27**	**0.15**	**Total**	**48.4**	**5.2**	**46.93‐49.87**	**−2.18**	**6.18**

Abbreviations: CI, confidence interval; PCS, pain catastrophization scale; Q, question; SD, standard deviation.

## 4 RELIABILITY

### 4.1 Internal consistency

Cronbach's α values for the whole and subsections of the Swa‐PCS are presented in Table 6.

**Table 6:** Cronbach's alpha (*α*) and ICC results for the Swa‐PCS as a whole, and sub‐sections.

PCS sections	Test mean (SD)	Retest mean (SD)	MD *t2−t1	*α*	ICC (95% CI)	SEM	MDC
Whole Swa‐PCS	49.12 (4.07)	48.4 (5.11)	−0.72	0.85	0.88 (0.82‐0.92)	3.38	7.82
Rumination	15.3 (1.15)	15.34 (1.19)	0.04	0.79	0.71 (0.57‐0.82)	2.06	4.76
Helplessness	22.64 (2.17)	22.46 (1.91)	−0.18	0.76	0.75 (0.631‐0.84)	0.95	2.19
Magnification	11.6 (0.75)	11.42 (1.17)	−0.18	0.69	0.67 (0.51‐0.79)	0.94	2.17

Abbreviations: ICC, intraclass correlation coefficient; MD, mean difference; SD, standard deviation; SEM, standard error of measurement; t2−t1, re‐test mean–test mean; α, Cronbach's alpha.

#### 4.1.1 Test‐retest reliability

ICCs and 95% confidence intervals for the whole and subsections of the Swa‐PCS are presented in Table 6.

#### 4.1.2 Ceiling and floor effects

Ceiling effect for the Swa‐PCS on time A was 48%, and for time B was 42%. There were no floor effects for either administration.

#### 4.1.3 Sensitivity‐to‐change: Measurement errors

**Figure 1:**
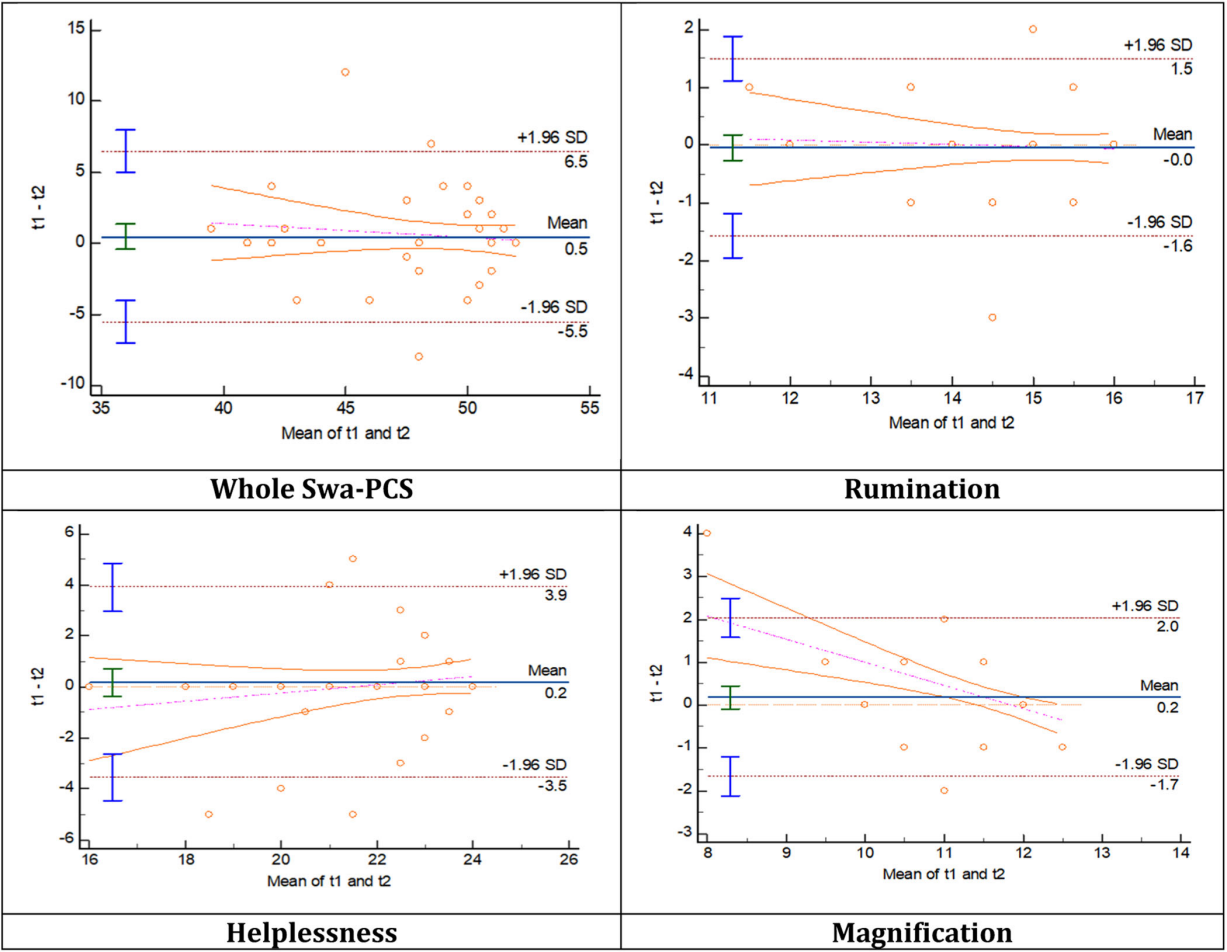
Bland‐Altman plots for the Swa‐PCS as a whole, and the sub‐sections (rumination, helplessness and magnification) showed good agreement between the two test scores, with approximately 95% of the differences lying within the 1.96 SD limits of agreement. This shows that there was no proportional bias between the test and retest scores. Swa‐PCS, Swahili version of the Pain Catastrophizing Scale.

SEm values for the whole and subsections of the Swa‐PCS varied between 0.94 and 3.38 and are listed in Table 5. Bland‐Altman plots were produced to show 95% absolute limits of agreement between the baseline and retest results (Figure 1). Bland‐Altman plots showed good agreement between the two test scores, with approximately 95% of the differences lying within the 1.96 SD limits of agreement. This shows that there was no proportional bias between the test and retest scores. Sensitivity‐to‐change was found to be satisfactory. The MDC of the Swa‐PCS indicated that a change of more than 7.82 points after a given intervention, would most likely not be due to measurement error. MDC values were calculated to establish the smallest change needed to reflect a true change and are listed in Table 6.

## 5 DISCUSSION

To our knowledge, this is the first study to report on the translation, cross‐cultural adaptation, nomological validity, construct validity, internal consistency, test‐retest reliability, sensitivity‐to‐change, ceiling and floor effects, and feasibility of the Swahili version of the PCS (Swa‐PCS) among refugees who are survivors of torture and/or war trauma living with chronic pain attending physiotherapy services in Kenya. The study found that for the translated and cross‐culturally adapted Swa‐PCS, internal consistency was satisfactory to good, for the whole and subsections respectively (range *α* = 0.69−0.85), with the lowest α found for the domain “magnification.” Test‐retest reliability for the Swa‐PCS was also found to be satisfactory to good (range ICC =  0.67−0.88). In addition, it was found that correlations between pain catastrophization and fear‐avoidance behavior scores were significant (*r* = 0.54, *p* < 0.01); ceiling effects were 42−48% with no floor effects, SEm values were between 0.94 and 3.38 and MDC were between 2.17 and 7.82. Confirmatory factor analysis confirmed that the Swa‐PCS had three factors which explained the majority of the variance.

Internal consistency for the Swa‐PCS, as a whole, was good (*α* = 0.85). These estimates are higher than the Spanish (*α* = 0.79), but on par with the French‐Canadian (*α* = 0.85), original English (*α* = 0.87), Catalan PCS (*α* = 0.89), the Korean versions (α = 0.90) and South African (*α* = 0.89−0.90) versions and lower than the German PCS (*α* = 0.94) versions.^18^, ^24^, ^26^, ^27^, ^29^, ^33^, ^57^ The homogeneity of the study sample should be considered in the interpretation of the resulting internal consistency. Although internal consistency reliability estimate is a property of a scale, it is highly affected by the construct's distribution in the sample. When a sample is this homogenous, tied together by common traumatic circumstances, the between variance is low and this could affect the internal consistency. Future studies should consider administering the Swa‐PCS among a more heterogeneous sample, for example among refugees experiencing pain but not seeing a physiotherapist, or where pain is the result of reasons other than torture or war trauma. In addition, the evaluation of internal consistency of the PCS as a whole measure may however not be theoretically correct,^58^ and was therefore calculated for each of the three subsections of the PCS. Internal consistency for the sections: helplessness, rumination and magnification of the Swa‐PCS ranged from *α* = 0.693 to 0.799, with the value for magnification being the lowest (*α* = 0.693). This result was however similar to that of the original, French‐Canadian, Catalan, Korean and German PCS, which reported Cronbach's *α* for the subsection “magnification” between 0.56 to 0.67.^18^, ^24^, ^26^, ^27^, ^29^ It is postulated that the lower internal consistency values observed in this domain is related to the fewer items contained in this subsection (3 items). However, according to Tavakol et al.^59^ low Cronbach's *α* may not always indicate construction issues with the tool; in the same way that higher values may not always reflect acceptable reliability.^59^ To make an accurate judgment, the characteristics of the scale that is, its length should be considered as well as the sample size.^59^ Further validation of the psychometric properties of the Swa‐PCS among larger sample groups is however warranted to confirm or negate this finding.

Test‐retest reliability of the Swa‐PCS as a whole was good (ICC = 0.878, 95% CI 0.822−0.923), and ranged between 0.672 and 0.750 for the subsections. This result was higher than the original English (ICC = 0.73), the Korean (ICC = 0.79), and the Catalan (ICC = 0.76); but on par with the Spanish (ICC = 0.84) and lower than the Chinese (ICC = 0.96) and South African versions (ICC = 0.89−0.91) of the PCS.^18^, ^24^, ^27^, ^33^, ^57^, ^60^ In this particular population, the variance in symptoms may also be influenced by many other factors such as being a refugee and having experienced trauma. Patients may be completely symptom free at the time of the retest or be experiencing an array of different symptoms, which might influence the test scores significantly and affect the reliability of the scale.^61^ However, the period between baseline and re‐testing was 2 weeks, which is typical of the period between consultations at similar study settings. One should therefore account for recall bias if the time period applied between the tests is too short or too long.^61^ Further testing is warranted.

Structural validity results showed that the Swa‐PCS had three factors which explained the majority of the variance in the data set, although RMSEA and CFI findings which were not within the acceptable ranges, and illustrated that it was not a good fit for the data.^49^ The number of factors for which most items had high loadings aligns with the findings reported for the original PCS which was divided into three sub‐sections helplessness, magnification and rumination.^18^ The Swa‐PCS can therefore assess the three identified domains of pain catastrophization among Swahili‐speaking survivors of torture and war trauma. It has to however be noted that item 12 was not the highest loaded in the expected component (helplessness), but was highest loaded in rumination. For this reason, it may be advised to move item 12 to rumination, as it might be a better fit. This result is similar to the German paper where item 12 was also loaded highest in the rumination component and it was suggested to move this item as well.^26^ Consideration in future studies for this suggestion should be given and structural validation of the Swa‐PCS should be further investigated.

The Swa‐PCS could not be correlated with a ‘gold standard’ which is a typical limitation faced by most PCS validation studies.^62^ Since no gold standard measure exists for pain catastrophization, a translated and/or adapted PCS is often correlated with related measures such as the FABQ.^62^ For this reason, in this study, the PCS scores and the FABQ scores were correlated at baseline and retest administration and nomological validation was established. It was found that on both occasions, the PCS and FABQ scores were significantly correlated (*p* < 0.05). The study found strong, significant and positive correlations between the PCS and FABQ scores (*r* = 0.538; *p* < 0.01). This finding is similar to the finding by Dover and Amar,^63^ who found that the PCS and FABQ‐total were significantly correlated as well (*p* = 0.005). No significant correlations were however found between PCS scores and pain (VAS) scores. This finding may however have been expected, since in chronic pain patients, typically pain and other symptoms vary daily and are influenced by a number of factors.^64^ Quantification of pain and other symptoms may therefore not be of much clinical use among patients suffering from chronic pain, and may in fact provide inaccurate information of a patient's disease state.^64^ This said, VAS scores are typically obtained in practice by physiotherapists and considered in the management plans of chronic pain patients. Physiotherapists, and other health professionals, should therefore reconsider the importance of all measures they seek to obtain from their patients and determine whether or not such measures are more detrimental than useful to the patient.

The high ceiling effects (48% and 42% at the two time points) found for the Swa‐PCS may indicate that the Swa‐PCS is not able to distinguish between respondents at the high end of the scale.^65^ Technically, it would be recommended that further research is needed to ascertain if additional changes to the Swa‐PCS would reduce the ceiling effects and render the Swa‐PCS better able to distinguish between either end of the scale. However, this said, it would not be unusual for most participants in this study to display high levels of pain catastrophization, therefore the high ceiling effect could be a genuine result of the study population's characteristics, and should be viewed as such. The high mean PCS scores may in fact be indicative of the current assumption that pain catastrophization plays a significant role in the mediation of chronic pain among survivors of trauma.^18^, ^19^

## 6 LIMITATIONS

Typically, cross‐cultural adaptation is conducted for populations who speak one language and who were born into the culture of interest.^43^, ^44^ However, in this study, although the participants all spoke Swahili, they were from different cultures outside that of the Kenyan context. Literature indicates that refugees may adopt the culture they find themselves in, and in this way the cultural adaptation of a particular instrument may become appropriate for them.^66^ However, it should not be forgotten that the current culture is not that of the participant and this in itself poses limitations and difficulty to extrapolate results. Generalizability of the results to other populations should therefore be made with caution. Moreover, the small study sample size can be attributed to the fact that the study population is a vulnerable and typically inaccessible group. Lower participant numbers and/or drop outs were therefore expected from the onset. However, it has to be cautioned that the small sample included in this study does limit the generalizability of the study results. Lastly, although all efforts to reduce bias were made, potential biases may have been introduced during the recruitment processes of the study. The participants were known to the principal investigator and may have only agreed to participate in the study for this reason. Future research should consider this as a potential source of bias.

## 7 IMPLICATIONS OR PHYSIOTHERAPY PRACTICE AND RESEARCH

With regard to implications for physiotherapy research this study does provide other researchers in comparable contexts with a logistical, yet preliminary, outline of how best to conduct validation studies in similar vulnerable populations. With regard to implications for physiotherapy practice, this study also provides clinicians with the opportunity to understand the importance of accurate outcome measurement among vulnerable groups such as refugees and torture or war trauma survivors who are more likely to suffer from chronic pain and other related conditions, and amongst whom measurement will be more difficult due to cultural differences in populations that migrate from one country to another. Although little remains known about this particular population, as physiotherapists and other health professionals become increasingly more involved in the management of refugees or displaced persons due to changes in the world, core competencies in the assessment and management of this population needs to be built and improved, and lessons learnt related to improving assessment and management within these vulnerable populations is best shared.

## 8 CONCLUSION

The current study findings indicate that, on a preliminary level, the translated and cross‐culturally adapted Swa‐PCS showed good internal consistency, test‐retest reliability and sensitivity‐to‐change and confirmatory factor analysis confirmed the three domains (helplessness, rumination and magnification) to assess pain catastrophization. Structural validation of the Swa‐PCS however requires further investigation. The Swa‐PCS scores were also related to fear‐avoidance behavior scores as expected. The Swa‐PCS is therefore feasible to be used among Swahili‐speaking refugees, who are survivors of torture or war trauma, living with chronic pain and attending physiotherapy services in Nairobi, Kenya. This paper does however also highlight some challenges faced which can inform future research. Further testing of the psychometric properties of the Swa‐PCS is warranted to confirm these findings and recommendations.

## AUTHOR CONTRIBUTIONS

**Jepkemoi J. Kibet**: Conceptualization, investigation, project administration, resources, writing draft/final manuscript; **Joliana S. Phillips**: Conceptualization, formal analysis, methodology, resources, supervision writing draft/final manuscript. **Mariem C. Latrous**: Data curation, writing draft/final manuscript. **Hanan Khalil**: Formal analysis, methodology, software, validation, writing draft/final manuscript; **Linzette D. Morris**: Formal analysis, methodology, software, validation, writing draft/final manuscript.

## CONFLICT OF INTEREST STATEMENT

The authors declare no conflict of interest.

## TRANSPARENCY STATEMENT

Linzette D. Morris Morris affirms that this manuscript is an honest, accurate, and transparent account of the study being reported; that no important aspects of the study have been omitted; and that any discrepancies from the study as planned (and, if relevant, registered) have been explained.

## Data Availability

All authors have read and approved the final version of the manuscript. L. D. Morris has full access to all of the data in this study and takes complete responsibility for the integrity of the data and the accuracy of the data analysis. Data is available on request from the corresponding author.

## APPENDIX 1 Swahili version of the Pain Catastrophization Scale

Lengo la hojaji hii ni kupata fikra na hisia unazoweza kuwa nazo unapokuwa na maumivu. Kuna nafasi tano za majibu kwa kila swali, hata kidogo, kwa kiwango kidogo, kwa kiwango cha wastani, kwa kiwango kikubwa, na wakati wote. Kwa kila swali, weka alama ya X katika nafasi inayolingana na kiwango cha hisia au fikra zako kuhusu kitu fulani. Tafadhali chagua nafasi moja pekee kwa kila swali. Tafadhali uliza mtafiti ikiwa huna uhakika kuhusiana na kinachoulizwa au unachopaswa kufanya.

**Table:**

		Weka alama ya X katika nafasi inayolingana na swali uliloulizwa				
Nambari	Fikra na hisia zako unapokuwa na maumivu	Hata kidogo	Kwa kiwango kidogo	Kwa kiwango cha wastani	Kwa kiwango kikubwa	Wakati wote
	Ninapokuwa na maumivu mimi huwa na wasiwasi kila kuhusu ikiwa maumivu hayo yataisha					
	Nikiwa na maumivu hukata tamaa					
	Nikiwa na maumivu hali yangu huwa mbaya kabisa na hufikiria kwamba sitapona					
	Nikiwa na maumivu huhisi vibaya sana na huhisi kwamba sitaweza kuhimili					
	Nikiwa na maumivu huhisi kwamba siwezi kuvumilia tena					
	Nikiwa na maumivu huogopa kwamba maumivu hayo yataongezeka					
	Nikiwa na maumivu hufikiria kuhusu matukio mengine machungu					
	Nikiwa na maumivu hutaka kwa haraka maumivu hayo yaniondokee					
	Nikiwa na maumivu hufikiria kuyahusu maumivu hayo kila mara					
	Nikiwa na maumivu hufikiria kila mara kuhusu jinsi inavyouma					
	Nikiwa na maumivu hufikiria vibaya sana kila mara kwamba maumivu hayo yanapaswa kuisha					
	Nikiwa na maumivu hakuna kitu ninachoweza kufanya kupunguza makali ya maumivu hayo					
	Nikiwa na maumivu huwa na wasiwasi sana kwamba kitu kibaya kinaweza kufanyika					
